# The Bill to Legalize Dissection, Its Veto

**Published:** 1881-11

**Authors:** 


### THE BILL TO LEGALIZE DISSECTION—ITS VETO.

---

It has always been regarded a most difficult matter to induce the Legislature to pass a bill for the purpose named in the heading. The profession, therefore, was agreeably surprised to learn that the General Assembly, just before its adjournment, had enacted such a law by a large majority in each house. Much praise was accorded Dr. Mathews, the member from Hart county, for urging the matter so diligently and successfully. A number of the physicians of this city presented him with a handsome testimonial of their appreciation. In fact, a considerable amount of jubilation, and no inconsiderable amount of champagne, was indulged in over his success. But, after all, the chicken was welcomed rather early, for it was not entirely hatched. No one dreamed the Governor would interpose his veto; but nevertheless, His Excellency did so interpose.

In point of fact, however, we doubt if so much was really lost to the profession by the veto as is generally thought. The sources designated in the bill, hedged by the limitations put around them, would probably furnish an altogether inadequate supply of material for the purposes of teaching.

The bill authorizes the proper authorities to deliver, and the colleges to receive, unclaimed bodies of persons dying in hospitals, prisons, etc., as must be interred at public expense; provided the remains shall not have been interred, or desired for interment by any friend or relative within twenty-four hours after death; or shall be so delivered against the consent of any relative or friend, if such is known to exist; and, provided, that the body of any one detained on suspicion of crime, or of any one who may have expressed a desire, in their last illness, that their body be interred, shall not be so delivered; and, provided further, that in case any such remains be subsequently claimed by any friend or relative, it shall be surrendered to him or them.

It will be seen that the person desiring the remains is not even required to express a purpose of burying them at private expense. In fact, if the provisions of the bill were faithfully carried out, but little real or immediate benefit, we believe, could be derived from it. But this does not palliate the blunder of the veto. It does not in the least mitigate the Governor's disregard of the wishes of the medical profession or the needs of science. The bill was a step in the right direction, and what of benefit it conferred, the profession was entitled to. No possible harm could have resulted. It is a shame and reproach upon the State that she should demand, by provision of law, that physicians shall possess certain skill, and yet, to the last degree, refuse them facilities for acquiring it. But the Governor kindly relieves the State of the onus by transferring it to his own shoulders.

We must confess that we scarcely have patience to write upon the matter. That a Governor who puts forward some claim to intelligence, and who is rather boastful of his interest in advancing science and education, who is trustee of an honorable medical college, should be guilty of such a veto, is almost beyond comprehension. With all the Governor's professions, we doubt if he has altogether escaped the relics of prejudice and superstition.

So much were we at a loss to surmise the reasons for the veto that we called at the Executive office to ascertain them. The Governor was absent; the secretary was not able to furnish them; the bill had been returned without endorsement or comment. We heard it stated that the

Governor thought the time allowed the friends for claiming the body of deceased was too short. If it can not be known within twenty-four hours that deceased has some friends or relative, it would probably not be known at all. If a body must be kept several days before embalming, the physician does not want it. This is the flimsiest of pretexts for a veto, as even a careless reading of the provisions quoted will show. Ample provision is made for the delivery of the body to the claimant, even at any time after the twenty-four hours have elapsed.

The general belief, so far as we have heard it expressed by physicians, and the one to which our knowledge of the Governor's methods gives color, is that the veto was made with an eye to his political future. There has been no more fruitful field of demagoguery than the convict question. It was one that was most successfully used in the last gubernatorial canvass; it is one that a certain class of aspiring politicians constantly hug to their bosoms. There is no surer way of securing the votes of the ignorant classes than appealing to and inflaming their prejudice on the chain-gang question. A pitiable sight would it be to see a measure for promoting medical science dragged into politics; but the approval or disapproval of such a measure as this can be used with tremendous effect before an audience of ignorant voters.

